# Meta-Analysis: A to Z for Healthcare Professionals

**DOI:** 10.7759/cureus.80846

**Published:** 2025-03-19

**Authors:** Mashael Al-Namaeh

**Affiliations:** 1 Clinical Research, Clinical Virtual Research Center, Wayne, USA

**Keywords:** egger statistical test, forest plot, funnel plot, meta-analysis, risk ratio, standard mean difference

## Abstract

Meta-analysis is a powerful tool in evidence-based medicine, allowing researchers to synthesize findings from multiple studies to enhance clinical decision-making. This technical report provides a step-by-step guide on conducting a meta-analysis using JASP (Jeffreys’s Amazing Statistics Program, University of Amsterdam, Amsterdam, Netherlands), an open-source statistical software. Two case studies illustrate its application: (1) an analysis of the association between serum vitamin D levels and intraocular pressure in primary open-angle glaucoma (POAG) and (2) an evaluation of the relationship between cardiovascular mortality and open-angle glaucoma (OAG). The study follows PRISMA guidelines and employs a random-effects model to account for heterogeneity. Results indicate no significant association between vitamin D and POAG (p = 0.122), while the cardiovascular disease-glaucoma meta-analysis shows high heterogeneity (I² = 99.54%). These findings highlight the challenges of interpreting meta-analyses with variable datasets. This report serves as a practical resource for researchers and clinicians, providing essential tools to conduct meta-analyses effectively and apply them in healthcare research.

## Introduction

The best available evidence in the medical literature is evidence-based medicine from meta-analysis, which offer a more accurate and less biased estimate of a clinical issue [1,2]. Meta-analysis is an objective, quantitative synthesis of research findings, characterized as a statistical method for integrating data from multiple studies addressing the same topic [3]. It is crucial to note that a meta-analysis may only be applied to studies where included research articles have comparable findings and the outcome measurements are the same [4]. Meta-analysis is guided by eight crucial phases of methodology: (a) research question, (b) research design, (c) literature review, (d) exclusion and inclusion criteria, (e) quality evaluation, (f) data synthesis, (g) writing and publishing, (h) updating your review [4]. The present study will use the relationship between serum vitamin D levels and primary open-angle as an example.

Meta-analyses can be performed using a few different software tools. In this meta-analysis, JASP v. 0.18.3.0 (Jeffreys’s Amazing Statistics Program, University of Amsterdam, Amsterdam, Netherlands) will be used. JASP has been employed for two purposes: Firstly, the University of Amsterdam supports this statistical analysis software, which is free and open-source [5]. Cost is a concern for some researchers and students; as research expenses rise, this tool can help you perform your meta-analysis more easily with no additional cost. (2) Secondly, it is made to be user-friendly and recognizable to SPSS users. Finally, users can download this program at any time and use it at work or at home.

In this article, two outcomes data will be used to demonstrate how to utilize the JASP software to perform dichotomous outcomes (such as the risk ratio (RR)) and continuous outcomes (such as the standard mean difference (std. MD)). The effect measures are either ratio measures such as RR or different measures such as std. MD [6]. 

The fundamental concepts and steps of conducting meta-analysis in clinical practice and research will be reviewed in this article.

## Technical report

How to perform a meta-analysis:

Step 1

Identification of Research Question and Gathering Data

Firstly, identify the relevant research question and gather data from various databases. Note: For our research question, we used PubMed and clinicaltrials.gov in this case.

Example: A Meta-Analysis of the Association between Serum Vitamin D Level and Intraocular Pressure in Primary Open-Angle Glaucoma

Research question: What is the relationship between serum 25-hydroxy- vitamin D (25(OH)D) levels and intraocular pressure in patients with primary open-angle glaucoma (POAG)? 

Gather data:A literature search was conducted using the PubMed database and clinicaltrials.gov. PubMed publications up to November 17, 2023, were searched for this study.

Keywords, Identification of the Meta-Analysis Guidelines, Inclusion Criteria, Flowchart Diagram

Determine the keywords and the identifying meta-analysis guidelines: glaucoma, vitamin D

Identification of the meta-analysis guidelines: Preferred Reporting Items for Systematic Reviews and Meta-Analyses (PRISMA) guidelines and criteria were adhered to in this meta-analysis study. The following keywords were used in PubMed research “Vitamin D” AND “POAG”. The selected studies applied no language or other restrictions as long as the article was translated into English.

Decide the inclusion criteria: All studies had to fulfil the subsequent requirements for inclusion (Table 1).

**Table 1:** The inclusion and exclusion criteria

	Inclusion Criteria	Exclusion Criteria
1	The investigation involved random sampling or cluster sampling	The investigation involved nonrandom sampling
2	The study was published in English	The study was not published in English
3	Paper scored at least six based on the Newcastle-Ottawa Quality Assessment Scale (NOS) for retrospective cohort studies and prospective cohort studies	Paper scored less than six based on NOS scale
4	The full text of the article was accessible	The full text of the article was not accessible
5	The subjects were human	Nonhumans/rodents or non-rodent experiment
6	Two or more comparison groups (glaucoma and control groups) were included	Only glaucoma group data were included
7	Both genders were recruited for both groups	Only one gender was recruited
8	Healthy subjects were recruited for the control group	No control group were recruited
9	A laboratory assessment of serum or plasma vitamin D levels were conducted	Vitamin D levels were not conducted

Draw your flow chart diagram: we identified 63 peer-reviewed studies and two clinical trial studies.

Step 2

Determine the Outcomes of the Study

The question of this meta-analysis was the potential correlation between vitamin D levels of POAG patients and controls. The outcome was vitamin D serum level in POAG patients. The methodological quality of the included observational studies was assessed using a modified version of the Newcastle-Ottawa Scale (NOS). The NOS has eight items in total and three separate subscales, with a maximum possible score of nine overall in a case-control study. We regarded a study with a score of ≥6 as high quality as there isn’t a consensus score that indicates what constitutes a high-quality study. A study with a 6-9 score rating has a high quality, a 3-5 rating has a fair quality, and a 0-2 has a poor quality. We evaluated eight studies, and the mean score was 6.67. The studies were done solely by one independent reviewer (MA).

The overall treatment effect was calculated and the study weight for each study was calculated. Due to the larger sample size of some of the studies, the study weight was calculated and the “true effect” for each study is shown. The larger sample size provides more information than a small sample size.

Determine the Null Hypothesis

The std. MD was computed in each study protocol to distinguish the association of vitamin D between healthy subjects and POAG patients. The random effect model was used to obtain the pooled std. MD. For each pooled std. MD, the analogous Z test was used to test the null hypothesis. if p <0.05 indicates that the difference between the two groups is statistically significant, the null hypothesis is rejected. The study heterogeneity was assessed with the Cochran Q and I^2 ^statistics.

Step 3

Firstly, perform the meta-analysis test and check between-study heterogeneity using the Cochran Q test and I^2^.

Note: Use a statistical fixed model if the heterogeneity is low, and a statistical random model if the heterogeneity is high. Because of the significant level of heterogeneity in this instance, we employed a random statistical model. 

• Gather all your data in Microsoft Excel file (Microsoft Corp., Redmond, WA).

Note: JASP was used for meta-analysis computations and plotting. The data was saved in Excel as CSV to be able to import the data into JASP software. Make sure that the file is saved as CSV using Excel software. 

• Open JASP software.

• Click on the “Main Menu” button, “Open”, “Computer”, “Browse”, and “Meta-analysis 1”.

Note: Importing data from an Excel file saved as CSV should allow you to have all of your data in the JASP program. In this case, we have 16 columns and 6 rows including Trial Number, Author, Year, Mean Vitamin D Glaucoma Group, SD (Glaucoma group), N, Mean Vitamin D Control Group, SD, N, TYPE of Glaucoma (Control group), Vitamin D Unit (ng/ml), Std. MD, Stderror, 95% CL Lower and Upper Range of Vitamin D, Variance, Weight of the Study).

• Click on “Meta-Analysis” and “Classical Meta-Analysis”.

• Fill effect size by clicking on “Std. MD”, Fill Effect Size Standard Error by clicking on “stderror”.

• For the Method, click on “DerSimonian-Laird method”, and fill in the study labels by clicking on “Author”.

• Under the Statistics section, under the regression Coefficients, click on “Estimates” and “Confidence intervals”, interval “95.0%”, under the model fit, clock on “Forest plot” and “Funnel plot”, and under the residuals Model click on “Residual parameters”.

• Under the Plots section, click on “Trim-fill analysis”.

Secondly, determine if you will use a fixed or random effect model and perform the forest plot.

In this case, we used the Std. MD as our measure. The size of the overall intervention effect was estimated by calculating the weighted average of the Std. MD between the groups in each study, and forest plots were created using the random-effects model. We used a random effect model in this case because the sample size is small.

Heterogeneity was assessed using the Q-statistic method (x^2^ and I^2^). For the heterogeneity qualitative interpretation, I^2 ^values of at least 50% were considered to reflect considerable heterogeneity, while values of at least 75% indicated large heterogeneity, as per the Cochrane Handbook. 

Step 4

Check publication bias using a funnel plot, Egger’s regression, and the trim and fill method.

Publication bias was evaluated using both a graphical funnel plot, Egger’s statistical test, and the trim and fill method using JASP software.

Note: The software used is JASP. Meta-analysis version 0.18.3.0 was used for statistical analyses. 

Step 5

• Present meta-analysis results based on PRISMA.

• Statistical analyses: verifying the preliminary analysis in the results window of JASP.

Step 6

Write the conclusion of the meta-analysis.

Examples of meta-analyses

Selection and Characteristics of the Study

**Figure 1:**
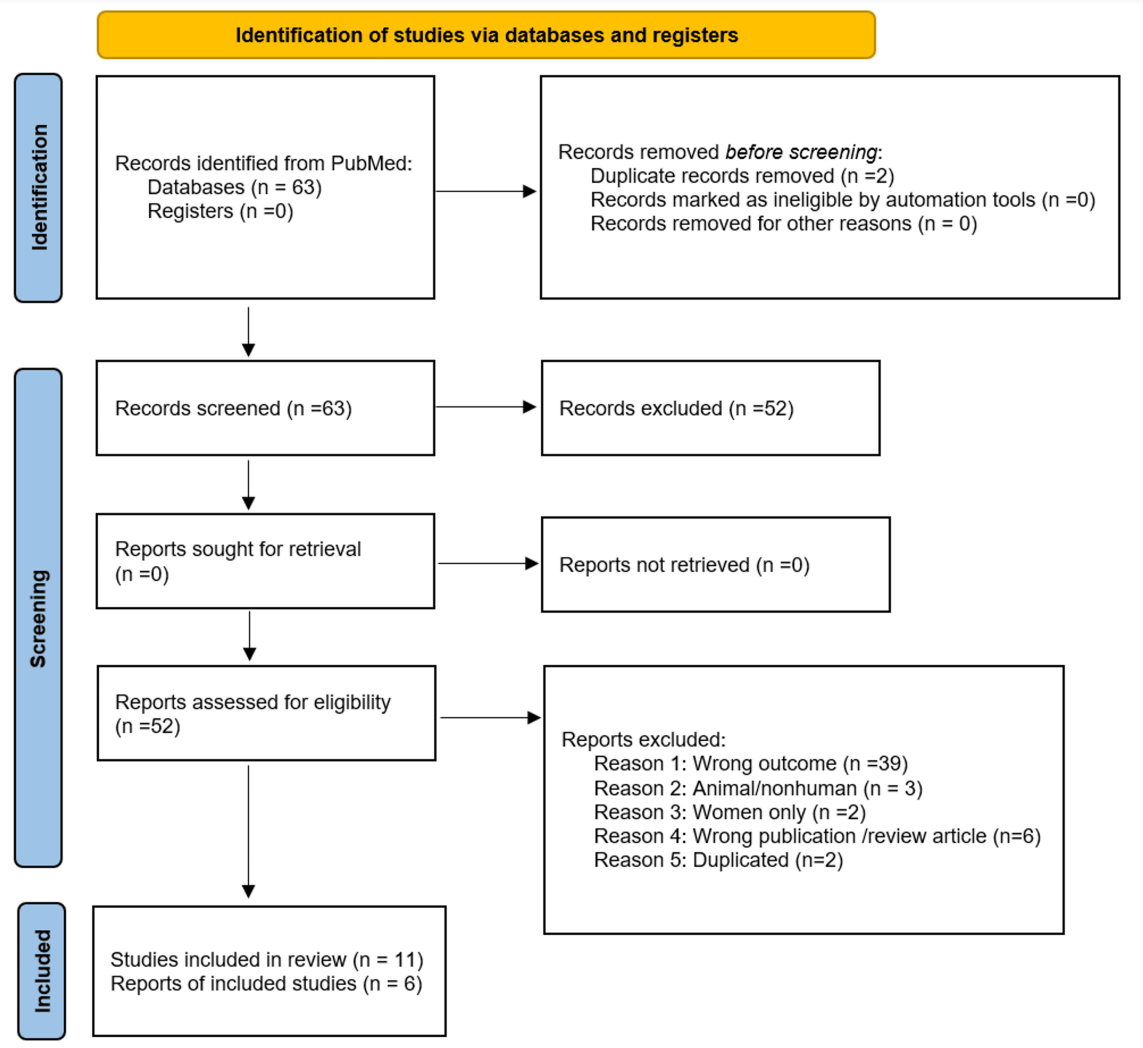
Flow chart presenting the selection of eligible studies

PubMed searches resulted in 63 articles. The titles and abstracts from the studies were evaluated, 52 were excluded (Figure 1), and 11 eligible studies were assessed for the systematic review and meta-analysis study. Six articles were retained for the quantitative eligibility primary outcome [7-12]. The flow chart presenting the selection of eligible studies is summarized (Figure 1) [13]. The characteristics of the included studies are summarized (Table 2). All the enclosed studies included a placebo group and a glaucoma patients’ group.

**Table 2:** Characteristics of included studies Newcastle-Ottawa Scale (NOS)

AUTHORS	STUDY ID	N	DESIGN	GLAUCOMA GROUP				CONTROL GROUP				NOS SCORE
				Age (years)	Female (%)	N	Vitamin D ng/mL mean ± SD	Age (Years)	Female (%)	N	Vitamin D ng/mL mean ± SD	
Yoo et al., 2014 [7]	1	5684	Cross-sectional (retrospective); South Korea	63.3 ± 10.7	46.90	290	18.10 ± 6.50	60.4 ± 10.1	57.00	5394	18.7 ± 6.6	7
Goncalves et al., 2015 [8]	2	314	Case-control (prospective); France	75.1 ± 8.5	42.00	150	17.16 ± 10.28	73 ± 7.9	59.80	164	19.76 ± 11.8	8
Lv et al., 2016 [12]	3	144	Case-control (prospective); China	61.03 ± 2.75	45.07	71	26.37 ± 5.83	60.14 ± 3.03	60.27	73	30.43 ± 3.91	7
Atalay et al., 2019 [11]	4	110	Cross-sectional (prospective); Turkey	62.4 ± 11.5	51.90	77	15.7 ± 12.00	57.8 ± 12.4	63.60	33	20.83 ± 14.06	6
Cho et al., 2021 [10]	5	126	Case-Control (prospective); South Korea	64.36 ± 11.77	50.00	36	21.22 ± 12.18	52.10 ± 13.9	40.00	90	18.12 ± 9.55	6
Bokhary et al., 2022 [9]	6	52	Cross-sectional case-control (prospective); Saudia Arabia	58.26 ± 9.55	59.20	27	29.03 ± 12.72	58.64 ± 10.62	52.00	25	26.86 ± 11.61	6

Independent Studies of Bias Risk

Egger’s test and funnel plot analysis were used to evaluate publication bias. Utilizing the NOS, which rates studies on a scale of 0 to 9 based on the selection of the research studies, comparability between cases and non-cases, and the assessment of the outcome (Table 3) [14,15], the quality of the studies was determined. The statistical analyses were conducted using Microsoft Excel. One study scored 8, [8], two studies scored 7 [7,9], and three studies scored 6 [9-11].

**Table 3:** The NOS scale of each study Newcastle-Ottawa Scale (NOS). Every star (*) represents one point in the Newcastle-Ottawa (NOS) scale.

STUDY	SELECTION				COMPARABILITY	EXPOSURE			TOTAL QUALITY SCORE
Author	Is the case definition adequate?	Representativeness of the cases	Selection of controls	Definition of controls	Comparability of cases and controls	Ascertainment of exposure	Same method of ascertainment for cases and controls	Non-Response rate	
Yoo et al., 2014 [7]	*	*	*	---	**	*	*	---	7
Goncalves et al., 2015 [8]	*	*	*	*	**	*	*	---	8
Lv et al., 2016 [12]	*	*	*	*	*	*	*	---	7
Atalay et al., 2019 [11]	*	*	---	*	*	*	*	---	6
Cho et al., 2021 [10]	*	*	---	---	**	*	*	---	6
Bokhary et al., 2022 [9]	*	*	*	*	*	---	*	---	6

Overall Final Analyses

**Figure 2:**
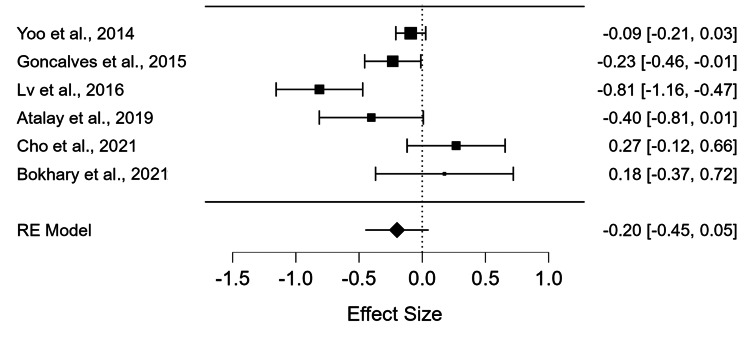
Forest plot for combined analysis of six studies utilizing the std. MD between the POAG group and the control group Yoo et al., 2014 [7], Goncalves et al., 2015 [8], Lv et al., 2016 [12], Atalay et al., 2019 [11], Cho et al., 2021 [10], Bokhary e al., 2021 [9] The x-axis shows the effect size and the the y-axis shows the effect size with the confidence interval (CI). It also shows the list of the studies. The size of the square shows the weight of the study. The diamond shows the combined results. Re Model= Random Effect Model

Thirteen studies on POAG were retrieved. Only six studies were included and met the eligibility criteria (n = 6430). In the combined results, the levels of vitamin D exhibited no difference between the POAG patients and controls (p = 0.122). (p = 0.122). The Std.MD with 95% confidence intervals (CIs) was -0.20 ng/dl (-0.45, 0.05) (Figure 2).

**Figure 3:**
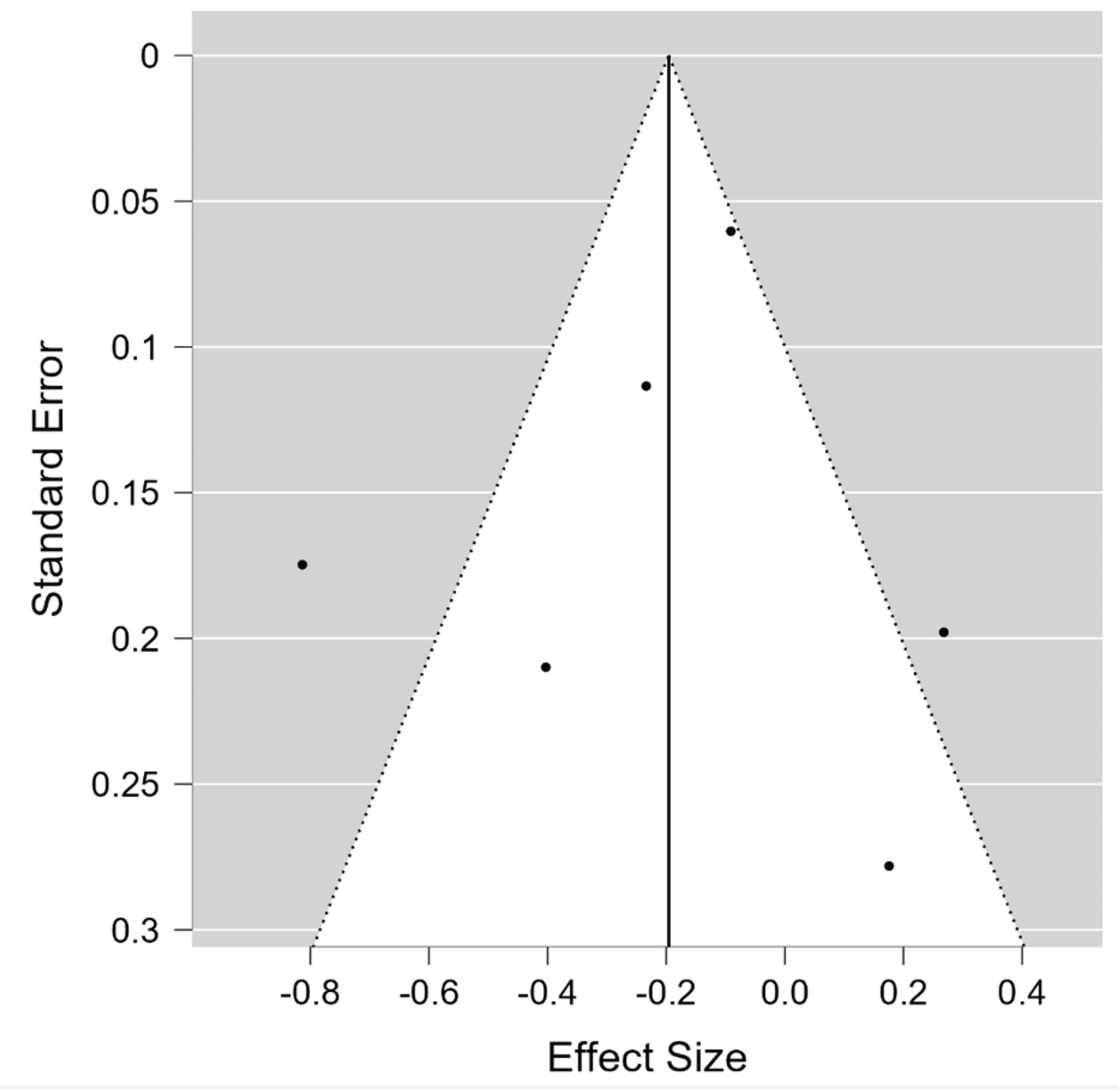
Funnel plot of standard error by Std. MD of the selected six studies showing symmetrical distribution of studies indicating absence of publication bias Yoo et al., 2014 [7], Goncalves et al., 2015 [8], Lv et al., 2016 [12], Atalay et al., 2019 [11], Cho et al., 2021 [10], Bokhary et al., 2021 [9]

No publication bias was found using the funnel plot and Egger’s test (P=0.754) (Figure 3). In this example, the I^2^ heterogeneity was high (I^2^= 78.441), which shows high heterogeneity between the studies.

**Figure 4:**
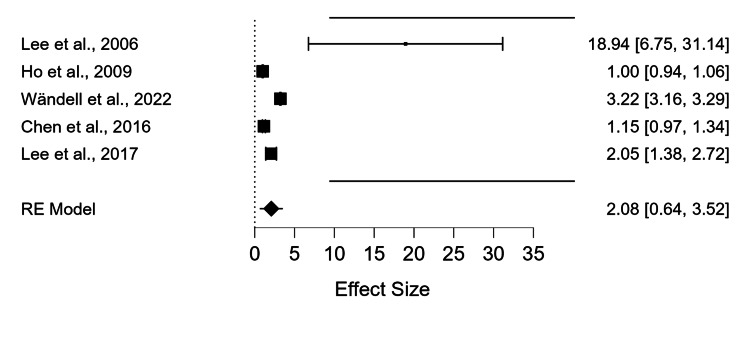
Forest plot for pooled analysis of five studies using the RR of the cardiovascular disease group in patients with open-angle glaucoma compared to the control group Lee et al., 2006 [16], Ho et al., 2009 [17], Wändell et al., 2022 [18], Chen et al., 2016 [19], Lee et al., 2017 [20]. The x-axis shows the effect size and the y-axis shows the effect size with confidence interval (CI); it also shows the list of the studies. The size of the square shows the weight of the study. The diamond shows the combined results. RE model=random effect model

**Figure 5:**
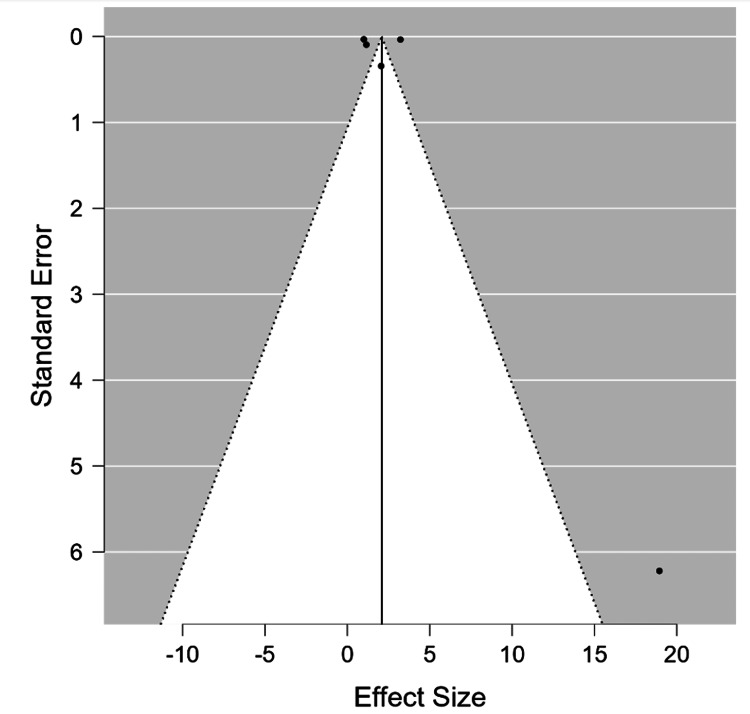
Funnel plot of standard error by RR of the selected five studies showing symmetrical distribution of studies indicating absence of publication bias Lee et al., 2006 [16], Ho et al., 2009 [17], Wändell et al., 2022 [18], Chen et al., 2016 [19], Lee et al., 2017 [20].

Another example to illustrate different examples of effect size is a study that used meta-analysis to determine the association between cardiovascular mortality and open-angle glaucoma. In this model, we used RR as an effect size rather than Std. MD. Five studies were considered in this study, with 24,681 participants in the open-angle glaucoma group and 1,738,790 participants in the control group. There is a statistical difference between the two cohorts, according to the analysis performed using the DerSimonian-Laird method to compare the RR. The overall RR is 2.08 with a 95% confidence interval of 0.64, 3.52, P<0.01). Significant heterogeneity was demonstrated (I^2^ value= 99.54 %, p<0.01). The I^2^ value shows that heterogeneity is due to the variability among studies rather than random chance. It also suggests inconsistent effects in magnitude and/or direction. The results showed that cardio-vascular ischemic diseases are not a risk factor for open-angle glaucoma. This is an illustration of a study with high heterogeneity (Figures 4-5). In conclusion, there is no indication of potential publication bias in the funnel plot (P=0.483). 

## Discussion

This meta-analysis included only case-control studies. It also highlights the fact that the available studies have a small number of cases that highlight the need for additional research. It also shows that heterogeneity was high (I^2^= 78.441) which shows high heterogeneity between the studies. The degree of variation in each study’s findings is known as heterogeneity [3]. Heterogeneity is necessary to identify the appropriate model to utilize. A fixed model is applicable when heterogeneity is low, while a random model should be used when heterogeneity is high. To identify and measure meta-analysis heterogeneity, Cochran’s Q test and I^2^ statistical tests were developed. The I^2 ^describes the percentage of variation among studies that is due to heterogeneity rather than chance [21]. However, the Q test cannot provide data on the degree of heterogeneity; rather, it merely provides meta-analysis with information on whether heterogeneity is present or absent [22]. It is important to note that despite the significant level of heterogeneity, this data still needs to be published because it highlights the significance of the need for additional research on this subject matter. Low heterogeneity may be defined as 0-40%, moderate heterogeneity as 30-60%, substantial heterogeneity as 50-90%, and high heterogeneity as 75-100% [23]. According to other scientists, more than 50% significant heterogeneity is present [24]. Another crucial step is deciding between fixed and random effects models. Fixed effects models may be considered if I^2^ is low and there are few variations across the studies [25]. Additionally, the random effect model should be considered if the Q value is significant P<0.01, which indicates that there is heterogeneity in the studies [26].

Typically, the funnel plot is a widely used graphical method to evaluate publication bias [27]. The funnel plot creates a symmetrical inverted funnel when publication bias is absent; asymmetry is indicative of publication bias [27]. Other techniques, such as Egger’s liner regression test [27], which measures funnel plot asymmetry using a natural logarithm scale of odds ratios, may be required if there are few studies because the funnel plot may be misleading. It is crucial to note that the” trim and fill” method adjusts the summary estimates for observed bias when asymmetry exists. According to this method, small studies are added or removed until funnel plot symmetry is reached by recalculating the funnel’s center before eliminating studies and substituting them with their missing mirror-image counterparts [28]. 

A modified version of the Newcastle-Ottawa Scale (NOS) was used to evaluate the methodological quality of the included observational studies because all of the investigations were case-control studies. 

The first systematic meta-analysis review evaluated the link between vitamin D and POAG. According to our findings, there is no link between vitamin D insufficiency and POAG, which is consistent with several published observational studies [18-20]. On the other hand, limited data suggest that vitamin D deficiency and glaucoma are positively correlated [14-17]. Our meta-analysis’s findings concur with the findings of Krefting et al., 2014 [29] and Lee et al., 2017 [20], showing that vitamin D deficiency was not linked to POAG; however, they differ from the findings of Kim et al., 2016 [30], Arar Vuković et al., 2016 [31] and Bokhary et al., 2021 [9] demonstrating that vitamin D levels were significantly lower in the POAG group compared to the control group.

The second systematic meta-analysis is another example of different effect sizes that were used to ascertain the relationship between cardiovascular mortality and OAG. The P-value could be influenced by a few outlier studies and a small sample size. If the sample size is small, as it is in our case, the P-value could be misleading. In this case, the confidence interval is easier to interpret. Therefore, in this case, we think that OAG is not linked to cardiovascular mortality, which emphasizes the need for more data. In this instance, the data is not statistically significant since the confidence intervals are easier to comprehend practically. 

One of the main advantages of a meta-analysis is that it yields a precise estimate of the effect size, with significantly increased statistical power, which is crucial when the primary study’s power is limited by a small sample size. When individual studies are inconclusive, they may also produce clear conclusive results. A meta-analysis also examines how the findings of many studies differ from one another and measure the heterogeneity of the studies. There are some recommendations that meta-analyses should be avoided if heterogeneity is high. I think even in cases when heterogeneity is considered high, we should emphasize that the findings of these individual studies differ in either direction magnitude, or both, which still calls for publication. One of the limitations of this study is that only one author performed the study. Finally, how to perform a meta-analysis is a very important tool to help physicians make clinical decisions.

## Conclusions

This technical report provides a structured approach to conducting meta-analyses using JASP, emphasizing its role in synthesizing clinical evidence. The vitamin D-POAG analysis found no significant association, while the cardiovascular disease-glaucoma meta-analysis exhibited substantial heterogeneity, underscoring the importance of careful statistical interpretation. These findings highlight the challenges of pooling heterogeneous data and the necessity for robust methodological approaches. By following the outlined steps, researchers can enhance the reliability of their meta-analyses and contribute to more informed clinical decision-making. Future studies should focus on improving study standardization and addressing sources of heterogeneity to strengthen meta-analytic conclusions in healthcare research.
